# Illness-related practices for the management of childhood malaria among the Bwatiye people of north-eastern Nigeria

**DOI:** 10.1186/1475-2875-4-13

**Published:** 2005-02-21

**Authors:** Oladele B Akogun, Kauna K John

**Affiliations:** 1 The Health Programme, Common Heritage Foundation, No. 1 Bishop St., Box 5124, Yola, Nigeria

## Abstract

**Background:**

A wide range of childhood illnesses are accompanied by fever,, including malaria. Child mortality due to malaria has been attributed to poor health service delivery system and ignorance. An assessment of a mother's ability to recognize malaria in children under-five was carried out among the Bwatiye, a poorly-served minority ethnic group in north-eastern Nigeria.

**Methods:**

A three-stage research design involving interviews, participatory observation and laboratory tests was used to seek information from 186 Bwatiye mothers about their illness-related experiences with childhood fevers.

**Results:**

Mothers classified malaria into male (fever that persists for longer than three days) and female (fever that goes away within three days) and had a system of determining when febrile illness would not be regarded as malaria. Most often, malaria would be ignored in the first 2 days before seeking active treatment. Self-medication was the preferred option. Treatment practices and sources of help were influenced by local beliefs, the parity of the mother and previous experience with child mortality.

**Conclusion:**

The need to educate mothers to suspect malaria in every case of febrile illness and take appropriate action in order to expose the underlying "evil" will be more acceptable than an insistence on replacing local knowledge with biological epidemiology of malaria. The challenge facing health workers is to identify and exploit local beliefs about aetiology in effecting management procedures among culturally different peoples, who may not accept the concept of biological epidemiology.

## Background

Malaria is a major cause of death among children in many parts of the world, despite the availability of simple and effective treatments [1] and is one of the main causes of morbidity and mortality in Nigeria [2,3]. Although, treatment often begins early and at home, a mother's inability to correctly recognize malaria has contributed substantially to child morbidity and mortality due to malaria [4]. In Tanzania, as in western Nigeria, studies show that mothers were unable to recognize severe malaria despite perceiving the signs and symptoms of onset of childhood malaria as including high temperature and loss of appetite [5]. Malaria control depends on many factors, some of which have not been studied at the level of rural communities, and in different cultural settings. An understanding of the mother's ability to appropriately manage childhood malaria in the home is crucial. In order to provide a a community with the capacity for dealing with the management of malaria in the home, a study of mothers' illness-related experiences in the management of childhood malaria was carried out among the poorly-served Bwatiye ethnic group of north-eastern Nigeria, the results of which are presented in this article.

## Methods

The study was carried out during the malaria season (May-September) of 1999 among the Bwatiye, a minority group living in six villages along the Benue valley in Adamawa State of north-eastern Nigeria. A multiple-stage approach consisting qualitative, quantitative and laboratory techniques was used.

### Local knowledge and help-seeking practices

In order to orient the data collection process, informal conversations were held with women and lay people in markets and health facilities about malaria illness experience within communities, the method of recognition and help-seeking behaviour. On the basis of received information, key informants were identified and selected (medicine vendors, folk healers, health-workers) and formally interviewed about local knowledge and practices regarding malaria in children. The information was used for designing a cross-sectional household survey questionnaire. Households with children between 9 and 60 months in six Bwatiye villages were identified with the assistance of community health personnel. The study objectives and procedures were explained to household heads and mothers of children that were eligible participants in the study. It was emphasized that anyone was at liberty to decline participation and that those participating were also free to withdraw at any stage of the study. The pre-tested cross-sectional survey questionnaire was administered in the Hausa language to 186 mothers in their homes. Information on the mother's demographic, educational and parity status was collected. Mothers were also asked about symptoms used for recognizing and classifying malaria and to describe their help-seeking practices when a child has malaria. Previous history of child mortality was also recorded. The interviews were complemented with observations of fifteen mothers (with previous child mortality, first childbirth, and more than two children) identified during the interview process. The results were analysed and used for developing a group discussion guide.

Six group discussions, each consisting of eight carefully selected mothers of different ages. family settings (polygamous or monogamous) and religions were held.

Culturally appropriate explanations were further obtained from four key informants when the data had been analysed and a draft report written.

## Results

### Presumptive diagnosis

Most mothers (88%) refer to malaria as *zazzabi*, meaning "hot body" with intermittent episodes of cold shivers ("when the child wants to stay in the sun") accompanied by headache as the main symptom of malaria in their children. Diarrhoea and teething were also important (44.6%) besides dehydration, sweating and vomiting (33.3%), loss of appetite (30.6%) and persistent crying (29.6%). Only very few mothers (6.5%) use faint spells, restlessness, moodiness and withdrawal as symptoms. Only 9.7% mentioned convulsion as an important symptom of malaria in children. Several symptoms were combined, although "child feels cold but has hot body" is an omnipresent clue to presuming that a child has *zazzabi *(Table 1).

**Table 1:** Symptoms^1 ^used by Bwatiye mothers for the recognition of childhood malaria

Symptom	Age (years)			Parity (childbirths)			Previous child mortality		Education		**Total**
	<25	26–35	>35	1^st^	2nd-3rd	4^th ^or more	Yes	No	Literate	Illiterate	

No in sample (%)	54(29)	59(32)	72(39)	42(23)	63(34)	81(44)	36(19)	150(81)	139(75)	47(25)	186(100)
Hot body (>38**C**)	77.9	89.3	93.2	100	92.1	79.0	100	85.3	87.8	89.4	88.2
Diarrhoea/ enteric complaints	38.9	47.5	46.6	61.9	81.0	28.4	80.6	36.0	49.6	29.8	44.6
Vomiting	29.6	32.2	37.0	33.3	30.2	35.8	11.1	38.7	37.4	21.3	33.3
Loss of appetite	31.5	30.5	27.4	38.1	28.6	28.4	58.3	24.0	28.1	38.3	30.6
Persistent crying	38.9	32.2	20.5	47.6	15.9	30.9	19.4	32.0	20.9	55.3	29.6
Dehydration /sweating	37.0	33.9	32.9	35.7	31.2	35.8	66.7	26.7	38.1	23.4	34.4
Convulsions	9.3	10.2	9.6	9.5	9.5	9.9	5.6	10.7	8.6	12.8	9.7
Other ^3^	9.3	5.1	5.5	11.9	4.8	4.9	8.3	6.0	6.5	6.4	6.5

^1^Multiple responses allowed

^2^Vertical comparison only (based on number of responses)

^3^Faint spells, insomnia, Restlessness, moodiness, withdrawal, colds

While the number of childbirths and previous experience with child mortality were important factors in the recognition of malaria (Chisquare test, P > 0.05), age and education (Chisquare test, P < 0.05) were not. Association between age and number of childbirths on the one hand and age and child mortality on the other were significant (Chisquare test, P < 0.05). Young mothers had fewer children and fewer child mortality rates than the older mothers. Among first time mothers, 47.6% identified fever with persistent crying, an association unique to this group and used *zazzabi *as the major symptom (100%), while 79.0% of those with more than three child-births were content to presume malaria on noticing "hot-body with cold shivers" without any additional symptom. Mothers with more than three children (28.4%) and mothers with two or three children (81.0%) differed widely with respect to enteric symptoms.

A history of child death in the family plays an important role in a mother's approach to recognition of malaria. Surprisingly, education played a minor role in the recognition of childhood malaria. For mothers who had experienced a child dying, emphasis was placed on diarrhoea.

### Classification

Malaria was classified into two categories; the female and the male. The female form goes away after a couple of days without any particular treatment other than that provided by the household or in some cases the mother. When illness defies home remedies after several days, or deteriorates, it is considered to be the male form. However, should extra-community consultants become necessary, the illness is no longer regarded as *zazzabi *and "evil doers" are suspected to be using *zazzabi *to hide a more severe ailment.

Convulsions or any form of complications including hallucinations were not regarded as symptoms of malaria but manifestations of other problems that the local healer (*boka*) rather than the health facility could handle.

### Help-seeking behaviour

**Figure 1:**
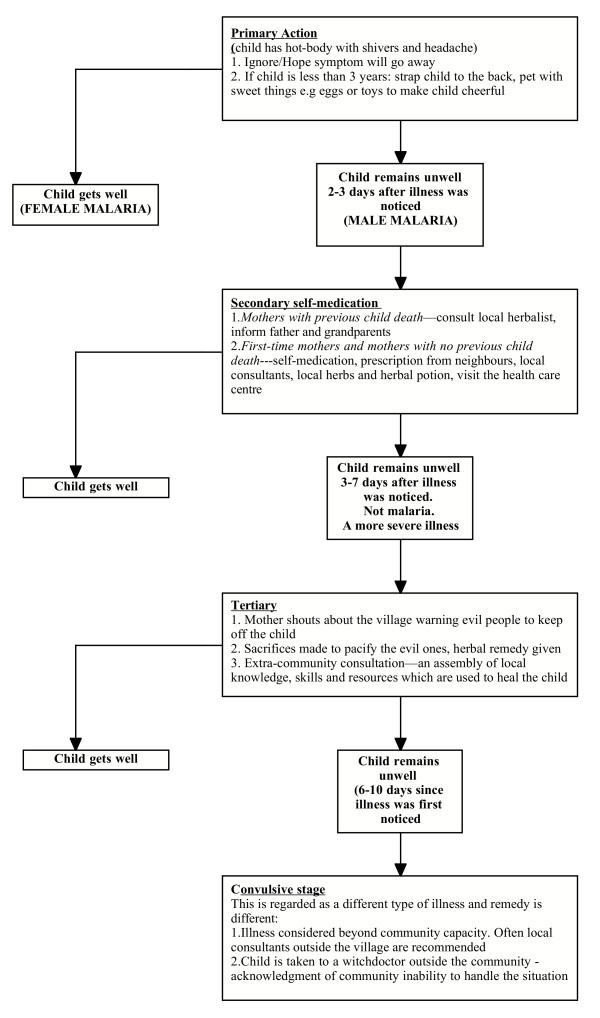
Flowchart of the sequence of help-seeking habits.

The sequence of action in progressive malaria infection among the Bwatiye is described in Figure 1.

Self-medication with herbs and herbal potions was the preferred option (60.2%) for most mothers (Table 2). However, young mothers were less inclined to use self-medication with herbs (53.6%) than older women (65.7%). They either waited for their husbands to take action (42.9%) or consulted an older woman or a mother-in-law (25.0%). On the other hand, those who had previously experienced child death (69.4%) preferred self-medication. Most mothers went to the health-care centre as the third line of action. Only few women without history of child death (11.3%) took their children to the health-care centre for treatment. Mothers often give traditional treatments for childhood convulsions and wait until fits cease before the next action. First-time mothers tended to consult their husband before anyone else. Multigravid women would first go to friends with previous experience with malaria in children to collect leftover tablets before informing the child's father or other male relatives.

**Table 2:** Help-seeking behaviour to malaria in children

	**Young women (< = 30)**	**Older women (>30)**	**Previous mortality**	**No history of child births**	**Total (%)**
**Sample **(N = 186)	84	102	36	150	186
Behaviour*					
Self-medication (with herbs, herbal potions):	45(53.6)	67(65.7)	25(69.4)	87(58.0)	60.2
Self-medication with antipyretics from market/shops	9(10.7)	15(14.7)	2(5.6)	22(14.7)	12.9
Treatment with drugs from health facilities	12(14..3)	8(7.8)	4(11.1)	16(10.7)	10.7
Treatment with herbs and drugs from market/shops	14(16.7)	16(15.7)	5(13.9)	25(16.7)	16.1
**If symptoms do not go away/get worse**					
Visit the herbalist	6(7.1)	48(47.1)	23(63.9)	31(20.7)	29.0
Visit the health facility/staff	8(9.5)	15(14.7)	6(16.7)	17(11.3)	12.4
Wait for husband before any action	36(42.9)	9(8.8)	2(5.6)	43(28.7)	24.2
Consult mother-in-law	21(25.0)	19(18.6)	2(5.6)	38(25.3)	21.5
Consult a more experienced friend or neighbour	13(15.5)	11(10.8)	3(8.3)	21(14.0)	21.9

* Simultaneous treatment with herbs and modern medicine is very common

Extra-communal effort at getting the child back to good health would then be made. Treatment which hitherto was confined to consulting immediate family members would now be extended to the wider community. Local herbalists and other neighbours then contribute to the management of the ailment on the basis of previous experience with similar or other illness. This is often done more as an attempt to show concern and solidarity rather than an understanding of the illness at hand. However if the child remains unwell, *evil doers *were regarded as blocking the effort to heal the child. The men would gather around the child to administer any medicine that may be brought by other community members. Women are excluded from the room at this stage. The "male form" of malaria is treated with both modern and traditional medicine of all sorts as precautions against spiritual dimensions to the illness. When convulsions set in, the child was immediately assumed to be severely ill and the treatment in any orthodox setting was most often ruled out. A furore of activities would begin to get the child out of the village to some other village, away from the reach of local enemies.

The most common anti-malarial drug used by the few mothers, who bought over-the-counter drugs, was chloroquine that was often administered at inappropriate dosage.

The 3-day course of chloroquine was never used in most cases mainly due to access and ignorance of the importance of full dose. Children with convulsions were taken first to the traditional healer and if it did not seem to be making any improvement the health-worker was consulted. Some health personnel thought convulsion was a different illness caused by cold and was best handled by a *boka*.

## Discussion

### Aetiological perspective and natural action

In Bwatiye, the term *zazzabi*, a Hausa word, implies an ordinary illness that does not kill. If death eventually results, it could not have been malaria, but some other cause.

As in other cultures, an attempt to differentiate between the severe and the mild forms of malaria has led to classification in order to determine at which stage a particular remedy would be required. While the Yoruba and Igbo are reported to have three classifications for malaria or fever episodes [6,7] the Bwatiye classified malaria into two as did the Kenyan tribe described by Munguti [8]. It has been demonstrated in many studies and for many illnesses, including river blindness and malaria [9-11], that many African communities are unable to perceive illness as a continuum of symptoms with one mild one giving way to a more severe one. Unlike the observations made by Linder [12] that the onset of fever in children often prompts mothers to seek immediate treatment, the natural primary action among the Bwatiye is to ignore the illness and hope it will go away. Persistent illness only elicits home remedy using available drugs or herbs within the household. Help is sought from the wider community (or from the local healer) only when the home remedy fails. Since there is a strong belief in the spiritual causes of illness, confidence and reliance in the local healer (the *boka*) is strong, especially among the older mothers. The local belief is that convulsions are not curable in health facilities and a sick child taken to another village, out of reach of the local enemies, is likely to survive. Most child deaths occur at this stage. In some cases, the child is taken to the health facility, often too late to be helped. This experience, which seems common, has created a strain between the health personnel and mothers, one accusing the other of negligence. The delay action of first-time mothers is interpreted as stemming from fear of rebuke by their husband, while third-time mothers being more confident, would rather wait to be sure it was not an ordinary illness. Third-time mothers are often older, more experienced, but also more entrenched in the local beliefs about malaria aetiology than the young mothers. Although they have more responsibilities, they are economically more independent and are able to take vital decisions with respect to their children with little reference to the husbands, not the case with the younger, inexperienced mothers. Unlike the younger ones, older and multigravid mothers are assisted by their older children who serve as child-minders and help with domestic and economic tasks. When drugs are used, the habit of sharing health resources in times of illness signifying solidarity contributes to non-compliance with management regulations. A mother stops administering drug as soon as the child seems to have improved in health and the remaining drugs are kept for another episode. The act of sharing has been exploited for the development of community management of childhood malaria in Uganda. The Bwatiye and other similar societies could benefit from this management approach.

The logical categorization of malaria as female (mild malaria) and male (severe), the interpretation of the class of ailment and its consequences, provide useful clues in the search for healing within the local milieu. Local consultants are useful allies in appropriately managing malaria or other febrile illnesses among the Bwatiye and perhaps other ethnic groups in north-eastern Nigeria.

### Implications for health education

The Bwatiye have an all-pervading belief that enemies often use *zazzabi *to cover up their evil deeds until it is too late for the affected person to seek a potent cure. This belief may not be ignored when drafting health education material. During post-analysis discussions, the women saw the logic of promptly administering antimalarial drugs at the onset of any febrile illness as a means of eliminating malaria and exposing any underlying evil that may not be malaria. The belief that malaria is incapable of killing a child without enemy intervention is a challenge to health educationists in this and other parts of Africa, where such beliefs prevail. These findings raise a number of points about what is appropriate in health education provision and empowerment for community health-care delivery. Should the development of educational materials be based on promoting biomedical aetiological theory of disease and should we insist that others accept it first, or should we work within the traditional understanding of disease transmission, which includes concepts of evil and ill intent?

The health authorities need to consider working within cultural epidemiological parameters if the programme objective is to reduce morbidity and mortality due to malaria. Insistence on acceptance of biomedical models of disease transmission belongs to another goal that may not be directly linked to reduction in morbidity and mortality.

## Conclusion

Until mothers are empowered with the capability to recognize and treat malaria, its impact on child mortality will continue unabated. The need is to educate mothers to suspect malaria first in every case of febrile illness and take appropriate action to rid the child of malaria to expose the underlying "evil". This is a more acceptable form of education than an insistence on replacing local knowledge with biological epidemiology of malaria. The challenge before health personnel is to identify and exploit local beliefs about aetiology in effecting management procedures among culturally different peoples irrespective of their acceptance of biological models of disease transmission.

## Authors' contributions

OBA conceived, designed, coordinated the implementation of the protocol, analysed and developed the manuscript while KKJ led the data collection team.
